# Annotations

**Published:** 1887-03-19

**Authors:** 


### Annotations.

The *Daily Telegraph*, in one of its highly spiced and "classically" garnished articles, calls the attention of its readers to the large amount of exceedingly useful work done by the London Dental Hospital during the last year. There are certain specialties and special hospitals which deserve to be commended, and this is one of them. From the point of view of the individual practitioner, "specialism" is a small and mind-narrowing occupation. The mere "specialist" is in danger of becoming a "poor creature" intellectually and humanly. But from the public standpoint there are decided advantages in specialism. A man who is drawing teeth every day of his life, though he may not become distinguished either as a saint or as a philosopher, can hardly help becoming a skilful tooth-drawer; and from him the public wants tooth-drawing, not philosophy. Specialism is in the ascendant, and tends to become increasingly so. The world likes its work well done. Dentistry is an art on which almost any man may become a critic without previous learning. Everybody knows when an aching tooth is cleverly extracted. Every woman can pass competent judgment on her neighbour's new set of teeth. We wish the dentists a continuance of their well-merited prosperity, but we counsel them all to enter boldly for their full medical and surgical qualifications. The world is prepared, on due cause shown, to recognise them as on a level with other professional men, but in order to gain and preserve a recognised position, they must possess the ordinary literary attainments of the educated classes as well as the mere mechanical skill of the makers, stuffers, and extractors of teeth.

**Teeth and their Guardians.**

Purveyors of sensational stories might prepare a choice feast for their peculiar portion of the reading public out of an incident which was brought before the notice of the House of Commons a few days ago. A poor woman was detained in the Asylum at Darenth for a considerable period, who does not seem to have been more insane than vast numbers of persons who are at large. She was merely the subject of some harmless illusions. She would not have been allowed to remain in the institution at all had there been any relatives or friends who were willing to take her in charge. She has now been transferred to other quarters. The incident serves to remind the public that sane persons may sometimes be found in these melancholy abodes. Not long ago a lady of some private means was incarcerated in a well-known asylum. She had made an attempt upon her life, and had thereupon been very properly placed under restraint. After two or three years she became quite sane, and then for the first time fully realised her dreadful position. Each time her relatives visited her she implored them with the most agonised entreaties to have her case inquired into with a view to her removal. Each time, relying on medical opinion, they disregarded her prayers. For months this continued, until the poor creature felt as if she would have welcomed even madness itself, as a deliverance from the horrors of her situation. At last her request was granted, and on the responsibility of an outside physician, who was no specialist, she was set at liberty. She has now enjoyed her freedom for nearly twelve months, requiring neither medical nor nursing supervision. She is in all respects perfectly sane. The moral of the story is this, that insanity is often of a temporary character, and that the insane should have the advantage of frequent visits from outside physicians. The border-land between sanity and insanity is so ill-defined, that a merely routine doctor, such as an asylum superintendent may occasionally become, utterly fails to notice when his patient has passed from the insane to the sane territory. A new pair of spectacles will often improve the sight.

**A Sane Woman in a Lunatic Asylum.**

"Cosmopolitan" thinks "England must be a peculiar country, London a more peculiar city, and the doctors of London the most peculiar of all." He falls ill, he says, and sends for a "physician." "A physician, sir," says the attendant; "which of them would you like—Sir Timothy Bolus, Sir Peppermint Mixture, or Dr. Rhubarb Pill?" "Oh, anybody will do; let me have the family attendant." Whereupon Dr. Calomel Powder is summoned and is speedily in attendance. Dr. Powder is very assiduous, very kind, and apparently intelligent and skilful. But still the patient gets a little worse, and appears to be in some danger. "Why don't you have a physician?" suggests a friend. "A physician—what do you mean? I have had a physician these ten days past." "Oh, yes, Dr. Powder; but a physician, you know—a physician." "But I tell you I have a physician. Dr. Calomel Powder is attending me." "Yes, yes, I know; but you should have a physician to see you along with Powder. Powder is not a physician, you know." "Not a physician! Why, then, in the name of fortune, what is he? Is he a kangaroo or a ringtailed monkey, or what? Has he no degrees?" "Degrees—Powder; certainly; he is a most respectable man; has got, I believe, M.D., and everything else *en règle*, and to my mind is a decidedly clever fellow; for anything I know, quite as good as Sir Timothy, or Sir Peppermint, or any of them; but it's the fashion, you know, to have a physician when you're more than usually ill." "Very well, send for one of them, by all means; but I should have thought that every duly qualified practitioner of physic could be defined in no other way so correctly as by calling him a physician. What is a physician? A man who practises physic. But England is undoubtedly a most mysterious and incomprehensible country."

**What is a Physician?**

